# Dental and composite resin discoloration induced by different hydraulic calcium silicate-based cements: two-year *in vitro* assessment

**DOI:** 10.1590/1678-7757-2022-0444

**Published:** 2023-04-28

**Authors:** Lucas Santos de JESUS, Tiago Lopes dos Santos REIS, Bernardo Guerra Mendonça de Melo MACHADO, Ana Luísa Monteiro da COSTA, Julia Menezes SAVARIS, Claudia Angela Maziero VOLPATO, Eduardo Antunes BORTOLUZZI, Cleonice da Silveira TEIXEIRA, Paula Cristina dos Santos Vaz FERNANDES, Lucas da Fonseca Roberti GARCIA

**Affiliations:** 1 Universidade Federal de Santa Catarina Centro de Ciências da Saúde Departmento de Odontologia - Endodontia Florianópolis SC Brasil Universidade Federal de Santa Catarina, Centro de Ciências da Saúde, Departmento de Odontologia - Endodontia, Florianópolis, SC, Brasil.; 2 Universidade do Porto Faculdade de Odontologia Departmento de Prótese Fixa Porto Portugal Universidade do Porto, Faculdade de Odontologia, Departmento de Prótese Fixa, Porto, Portugal.; 3 University of Louisville Department of Diagnosis & Oral Health Endodontics Division Louisville KY USA University of Louisville, Department of Diagnosis & Oral Health - Endodontics Division, Louisville, KY, USA.

**Keywords:** Hydraulic calcium silicate-based cement, Mineral trioxide aggregate, Color difference, Tooth discoloration, Composite resin

## Abstract

Few long-term studies assess the discoloration induced by hydraulic calcium silicate-based cement on dental structures. In addition, as far as we know, no long-term study has assessed the discoloration induced by these cement on composite resin.

**Objective:**

This *in vitro* study aimed to assess, during a period of two years, the discoloration potential of different hydraulic calcium silicate-based cements (hCSCs) on the enamel/dentin structure and composite resin restoration.

**Methodology:**

A total of 40 enamel/dentin discs were obtained from bovine incisors, and 40 composite resin discs (10 mm in diameter × 2 mm thick) were fabricated. A 0.8 mm-deep cavity was made in the center of each disc and filled with the following hCSCs (n=10): Original MTA (Angelus); MTA Repair HP (Angelus); NeoMTA Plus (Avalon); and Biodentine (Septodont). An initial color measurement was performed (T0 - baseline). After 7, 15, 30, 45, 90, 300 days, and two years, new color measurements were performed to determine the color (ΔE00), lightness (ΔL’), chroma (ΔC’), hue differences (ΔH’), and whiteness index (WID).

**Results:**

For enamel/dentin, the ΔE00 was significant among groups and periods (p<0.05). NeoMTA Plus had the greatest ΔE00. The NeoMTA Plus group had the greatest ΔE00 after two years for composite resin. Significant reduction in lightness was observed for all groups after two years (p<0.05). The most significant WID values were observed after 30 days for Biodentine (enamel/dentin) and MTA Repair HP groups (composite resin) (p<0.05).

**Conclusions:**

The hCSCs changed the colorimetric behavior of both substrates, leading to greater darkening over time. The Bi_2_O_3_ in the Original MTA seems relevant in the short periods of color change assessment.

## Introduction

Hydraulic calcium silicate-based cements (hCSCs) have been widely used in Dentistry as repair biomaterials due to their excellent mechanical, chemical, and biological properties.^1-3^ The precursor of this class of cement was the Mineral Trioxide Aggregate (MTA), initially developed as a retro filling material and as a root perforation sealing.^1,2^ However, due to its good features, its clinical application quickly expanded beyond these treatments.^1-3^

Despite the success achieved by MTA in several clinical situations, studies have reported that this hCSC causes discoloration of dental tissues in a short period of time.^4,5^ Therefore, since the introduction of MTA in the early 1990s, the cement composition has been modified to improve its physical-chemical properties and minimize the adverse effects on dental esthetics.^1-5^

The initial version of MTA (grey MTA) had a high amount of iron oxide (Fe_2_O_3_) in its composition,^6^ which led to intense crown discoloration, especially in the cervical portion of the tooth.^4,5,7^ Despite the introduction of a new MTA (white MTA) with a lower concentration of Fe_2_O_3_, it also promoted tooth discoloration.^4,5,7^ Many studies have suggested that the MTA radiopacifier, the bismuth oxide (Bi_2_O_3_), was responsible for tooth discoloration^8-10^ since this compound destabilizes when in contact with the sodium hypochlorite solution, which is used for root canal preparation.^9,11^ In addition, the amino acids in the collagen fibrils of the dentin organic matrix destabilize the Bi_2_O_3_, leading to a dark precipitate deposition.^10^ Despite the changes in the MTA composition to improve its esthetical performance, it still promotes some degree of tooth discoloration throughout time.^4,5,7-11^

Therefore, new hCSCs have been developed to overcome this limitation of MTA.^12^ The MTA Repair HP (high plasticity) (Angelus, Londrina, PR, Brazil) is an evolution of the original MTA cement, with improved rheological properties due to the addition of an emulsifying agent to the distilled water used for its manipulation.^13,14^ Additionally, the Bi_2_O_3_ was replaced by the calcium tungstate (CaWO_4_) as a radiopacifier.^13,14^ The NeoMTA Plus (Avalon Biomed Inc. Bradenton, FL, USA) is mixed with a water-based gel that results in good handling characteristics to the cement. Its radiopacifier is tantalum oxide (Ta_2_O_5_).^15,16^ Finally, the Biodentine (Septodont, Saint-Maur-des-Fossés, France) is claimed as a permanent bulk dentin substitute with improved mechanical properties compared to the original MTA.^17,18^ Its radiopacifier is the zirconium oxide (ZrO).^17,18^

Despite some success in avoiding tooth discoloration by hCSCs in short periods, it is a consensus among researchers that hCSCs promote changes in the color behavior of the dental structures in the long term, but the real mechanism of this discoloration is still uncertain.^4,5,7-11,19^ Możyńska, et al.^5^ (2017) have reported that some hCSCs have a high potential for hard tissue discoloration, whereas some others promoted a small color change, which was nearly invisible to the human eye.

In addition, little scientific evidence exists on the potential discoloration induced by hCSCs in resinous materials, such as bonding agents and composite resin restorations.^20,21^ Therefore, this *in vitro* study aimed to perform a two-year assessment of the discoloration potential of four different hCSCs (original MTA, MTA Repair HP, NeoMTA Plus, and Biodentine) on the enamel/dentin structure and composite resin restoration. The tested null hypothesis was that the types of cement would not induce color (ΔE_00_), lightness (ΔL’), chroma (ΔC›), and hue (ΔH›) differences on the enamel/dentin structure and composite resin, in addition to changes in the whiteness index (WI_D_).

## Methodology

### Experimental design

To determine the discoloration potential of hCSCs, a 4×7×2 experimental design was adopted, with the hCSCs (four levels), storage periods (7, 15, 30, 45, 90, 300 days, and two years in artificial saliva), and substrates (enamel/dentin and composite resin) as factors. The change in color parameters over time was considered a response variable. The sample size was calculated with the aid of the G*Power software (version 3.1.9.6; http://www.psycho.uni-duesseldorf.de/abteilungen/aap/gpower3/). The following parameters were used for calculation: α err prob = 0.5; power (1-ß error probability) = 0.80; and effect size dz = 0.5. The Wilcoxon signed-rank (matched pairs) statistical test was performed. The type of power analysis was set *a priori* (compute required sample size - given α, power, and effect size). The calculation was based on two-tails and a normal parent distribution. Based on this analysis, ten specimens were allocated to each of the experimental groups.

### Specimens preparation - enamel/dentin discs model

A total of 40 freshly-extracted bovine incisors aged approximately three years were donated by a local slaughterhouse. Considering the institutional guidelines of this research, ethical approval is unnecessary. The teeth were immersed in 0.5% chloramine solution for 48 hours for disinfection and washed under running water for 24 hours. Scalpels and gauze pads were used for periodontal ligament removal. After a rigorous clinical inspection at 4× magnification, teeth with signs of crown discoloration were discarded from the final sample.

A preliminary color measurement was performed using an intraoral colorimetric device (VITA Easyshade Advance 4.0; VITA Zahnfabrik, Bad Säckingen, Germany) to allow a proper control and uniform distribution of the bovine teeth within the experimental groups. The teeth crowns were cleaned with a prophylactic paste and washed with copious running water. After the drying process, the teeth were placed on a white background (standard calibration tile following the *Commission Internationale de l’Éclairage* - CIE - L*: 93.84, a*: -1.48, and b*: 3.76) and subjected to color measurement at the center of the crown buccal surface.^22^ A neutral color condensation silicone matrix (Zetaplus; Zhermack, Badia Polesine, Italy) was fabricated and coupled to the VITA Easyshade Advance 4.0 device tip to allow a proper measurement at the central area of the buccal surface of each bovine tooth. The color measurement was performed in triplicate for each tooth by a previously trained and calibrated operator. The VITA Easyshade Advance 4.0 device was also calibrated before each new measurement.

The coordinates L*a*b* were recorded at the end of color measurements and the mean values were estimated. Following the study by de Jesus, et al.^7^ (2021), the teeth were distributed into four groups (*n*=10) according to their colorimetric data to avoid significant variation among specimens from the same experimental group. For this, the data provided by the color measurements were correlated to the color scale guides (VITA Classical scale guide and VITA 3D Master guide) provided by the VITA Easyshade Advance 4.0 device. The experimental groups were formed with a balanced distribution of teeth with the same colorimetric references (e.g., three teeth color A2, three teeth color B1, two teeth color B2, and two teeth color A3 in the same experimental group).^7^

After this distribution, the crown of each tooth was sectioned at the cementoenamel junction using a double-sided diamond saw (Buehler, Lake Bluff, IL, USA) coupled to a metallographic cutter (Isomet 1000; Buehler) operating at 300 RPM under copious water-cooling. Next, a trephine drill (Neodent, Curitiba, PR, Brasil) was used coupled to a low-rotation device (MRS 400 model, Dabi Atlante, Ribeirão Preto, SP, Brazil) under abundant water cooling. Enamel/dentin discs with 10.0 mm in diameter and 2.0 mm-thick (±) were obtained from the central area of the buccal surface of the teeth crowns. The discs were then smoothed with a fine-grained diamond cylindrical drill (Komet, Savannah, GA, USA), coupled to a low-rotation device (MRS 400 model; Dabi Atlante) under water cooling. Next, the discs’ surfaces, enamel, and dentin, were polished with abrasive water sandpaper (Norton, São Paulo, SP, Brazil) in a decreasing sequence of abrasiveness (400, 600, and 1200 grit) until the enamel and dentin layers were 1.0 mm-thick each. The final thickness of the specimens was measured with a digital caliper (Digimess; Shinko Precision, Gaging, China).

**Figure 1:**
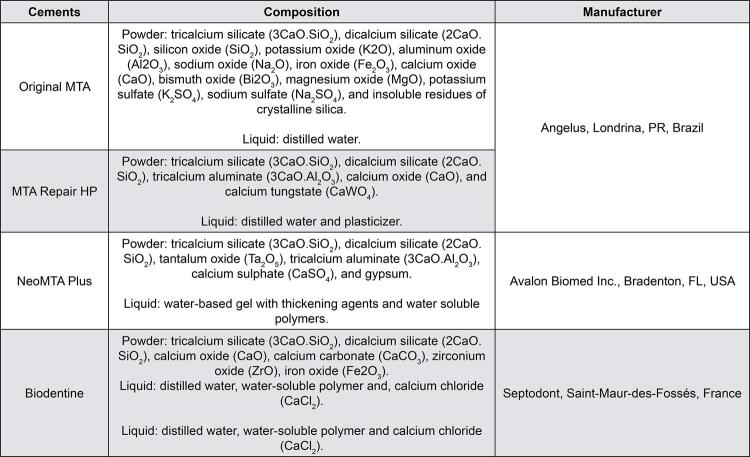
Hydraulic calcium silicate-based cements used in the study

A 0.8 mm-deep cavity was made in the center of the dentin disc surface with a 0.5 mm-diameter spherical diamond bur (KG Sorensen, Barueri, SP, Brazil) coupled to a high-speed device (PB model; Dabi Atlante) under abundant water cooling. To standardize the cavity depth, a composite resin stop was fabricated and placed at a distance of 0.8 mm from the cutter extremity of the bur. Then, the cavities were filled with the experimental hCSCs to be tested and distributed as follows (*n*=10): Original MTA group (positive control); MTA Repair HP group, NeoMTA Plus group, and Biodentine group (Figure 1). The hCSCs were strictly manipulated according to their manufacturers.

The enamel/dentin discs were stored for seven days in an environment with 100% humidity at 37°C to allow the setting of cement. Then, pre-etching of the dentin surface was performed with 37% phosphoric acid (Condac 37; Joinville, SC, Brazil) for 15 seconds, followed by washing with water and drying with absorbent paper. Two layers of adhesive (Adper Single Bond 2; 3M ESPE Dental Products St. Paul, MN, USA) were applied on the dentin surface for 20 seconds each and, then, air-dried for five seconds. The excess adhesive was removed with absorbent paper. The solvent was air-dried, followed by light curing for 40 seconds (FlashLite 1401; Discus Dental, Culver City, CA, USA - light intensity ≥1100 mW/cm^2^, wavelength between 460 and 480 nm). Afterwards, the cavities were restored with composite resin (color A3) (Z250; 3M ESPE Dental Products) using the incremental technique. Each layer of composite resin was light-cured for 40 seconds (FlashLite 1401; Discus Dental).

### Specimens preparation - composite resin discs model

A total of 40 specimens of composite resin (color A3) (Z250; 3M ESPE Dental Products) were fabricated in a Teflon mold (10.0 mm in diameter × 2.0 mm thick) by the incremental technique. The specimens were light-cured for 40 seconds (FlashLite 1401; Discus Dental) and, after removal from the Teflon mold, they were polished with abrasive water sandpaper (Norton) in a decreasing sequence of abrasiveness (320, 600, and 1200 grit). The thickness of the specimens was measured with a digital caliper (Digimess; Shinko Precision). Since the specimens were fabricated with an A3 color composite resin, the preliminary color measurement for a uniform distribution of the specimens based on their colorimetric data was unnecessary.

As described for the enamel/dentin discs, a 0.8 mm-deep cavity was made in the center of the composite resin discs, which was filled with the hCSCs to be tested (*n*=10). At the end of the hCSCs setting period, the cavities were restored with composite resin, following the same adhesive protocols previously described.

### Color measurement

After the enamel/dentin and composite resin discs were restored, an initial color measurement (T0 - baseline) was performed at the center of the enamel surface and at the center of the surface opposite to the cavity containing the hCSCs for composite resin specimens, following previous descriptions. Once again, a neutral color condensation silicone matrix (Zetaplus; Zhermack) was fabricated and coupled to the VITA Easyshade Advance 4.0 device tip for proper measurement at the central area of each specimen. The color measurement followed the CIE L*a*b* system. According to this system, the L* coordinate represents an object lightness, ranging from absolute black (0) to absolute white (100). The a* coordinate represents the green to red value rates (-90 to 70), and the b* coordinate represents the rate from blue to yellow (-80 to 100).^22^

At the end of the initial color measurement (T0 - baseline), the discs were stored in artificial saliva (Dermus Pharmacy, Florianópolis, SC, Brazil) to simulate the oral conditions and were kept in an oven at 37°C for two years. The artificial saliva (Dermus Pharmacy) was replaced every seven days. After 7, 15, 30, 45, 90, 300 days, and two years, new color measurements were performed to compare the L*a*b* standard mean values (baseline).

### Color, lightness, chroma, and hue differences calculation

The color (∆E_00_), lightness (∆L’), chroma (∆C’), and hue (∆H’) differences among the standard mean values (baseline) and the experimental periods of analysis (7, 15, 30, 45, 90, 300 days, and two years), were estimated using the following equation (CIEDE2000 system):^22-24^


$$\Delta E_{00}={\left[{\left(\frac{\Delta L^{\prime }}{K_{L}S_{L}}\right)}^{2}+{\left(\frac{\Delta C^{\prime }}{K_{C}S_{C}}\right)}^{2}+{\left(\frac{\Delta H^{\prime }}{K_{H}S_{H}}\right)}^{2}+R_{T}\left(\frac{\Delta C^{\prime }}{K_{C}S_{C}}\right)\left(\frac{\Delta H^{\prime }}{K_{H}S_{H}}\right)\right]}^{1/2}$$

ΔL', ΔC', and ΔH' represent the differences in lightness, chroma, and hue, respectively;

R_T_ is the rotation function used to explain the interaction between chroma and hue in the blue region;

S_L_, S_C_, and S_H_ are weighting functions;

K_L_, K_C_, and K_H_ are parametric factors adjusted for 1.^23^

The CIELAB-based whiteness index calculation

The whiteness index (WI_D_) of the standard mean values (baseline) and the experimental periods of analysis (7, 15, 30, 45, 90, 300 days, and two years) were calculated using the following equation (CIELAB system):^25^


$$WI_{D}=P\times a^{*}+Q\times b^{*}+R\times L^{*}$$

The coefficients P, Q, and R were based on visual observations performed in four psychophysical experiments (under both laboratory and typical clinical conditions),^26^ correlating the CIELAB system values close to reference white (L*=100, a*=0 and b*= 0) by positively weight low chromaticity (a* and b* values closer to 0) and high brightness values (L* values closer to 100).^25^

### Statistical analysis

The normal distribution of data was confirmed by the Shapiro-Wilk test. The level of significance was set at α = 0.05. Color (ΔE_00_) differences for enamel/dentin and composite resin groups were evaluated by Two-way ANOVA (hCSCs × time). The lightness (ΔL'), chroma (ΔC'), and hue (ΔH') differences were evaluated by repeated-measures ANOVA. The WI_D_ values were evaluated by One-way ANOVA, comparing the initial references of each group (7 days - Table 3) with the tested periods. Multiple comparisons were performed by *post hoc* Tukey’s HSD test (α=0.05). The statistical analysis was performed using SPSS 21.0 for Windows software (SPSS Inc., Chicago, IL, USA).

**Table 3:** Averages of whiteness index (WID) for enamel/dentin and composite resin groups and periods

Enamel/dentin	7 days	15 days	30 days	45 days	90 days	300 days	2 years
Original MTA	14.9 ± 6.5	17.8 ± 8.6	18.6 ± 3.1	19.2 ± 4.3	16.3 ± 5.7	15.1 ± 5.9	13.0 ± 3.7
MTA Repair HP	17.4 ± 3.7	18.4 ± 3.8	19.3 ± 3.4	16.1 ± 4.1	16.5 ± 3.1	17.6 ± 4.9	12.1 ± 4.8
NeoMTA Plus	14.9 ± 5.4	17.1 ± 5.8	17.4 ± 6.1	18.3 ± 6.0	15.9 ± 3.4	13.1 ± 5.9	13.8 ± 4.0
Biodentine	18.0 ± 2.8	19.6 ± 4.5	20.0 ± 6.5	19.4 ± 4.3	17.7 ± 5.1	13.8 ± 9.9	10.7 ± 9.1
**Composite resin**	**7 days**	**15 days**	**30 days**	**45 days**	**90 days**	**300 days**	**2 years**
Original MTA	17.8 ± 2.5	19.5 ± 2.4	19.5 ± 2.6	18.5 ± 2.7	18.9 ± 2.4	17.2 ± 1.7	17.2 ± 2.6
MTA Repair HP	17.0 ± 2.1	18.9 ± 3.9	19.6 ± 1.8	18.3 ± 1.9	16.8 ± 3.3	16.7 ± 2.4	15.8 ± 3.4
NeoMTA Plus	14.3 ± 2.2	16.8 ± 2.4	15.6 ± 1.8	17.1 ± 1.7	15.3 ± 1.8	13.6 ± 1.9	15.2 ± 3.9
Biodentine	13.2 ± 2.5	13.7 ± 2.4	14.4 ± 2.2	16.4 ± 1.9	13.7 ± 1.6	12.6 ± 2.9	11.1 ± 4.1

## Results

**Figure 2:**
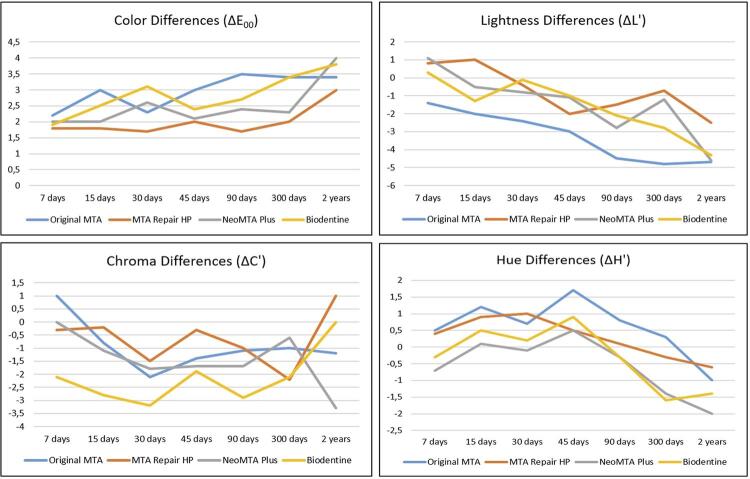
Graphic representation of color (ΔE00), lightness (ΔL'), chroma (ΔC'), and hue (ΔH') differences for enamel/dentin groups and periods

**Figure 3:**
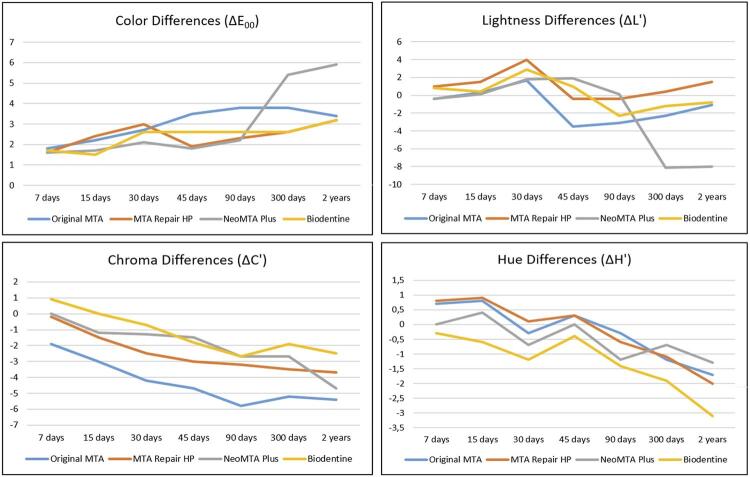
Graphic representation of color (ΔE00), lightness (ΔL'), chroma (ΔC'), and hue (ΔH') differences for composite resin groups and periods

**Figure 4:**
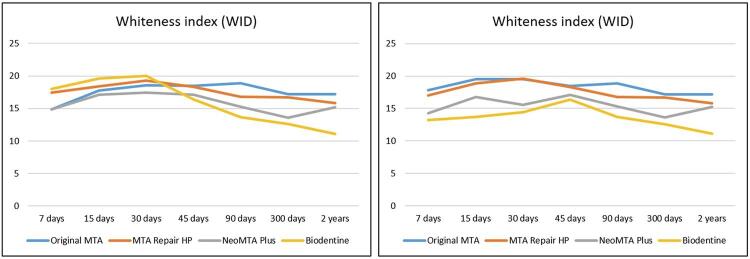
Graphic representation of whitening index (WID) differences for enamel/dentin (left) and composite resin (right) groups and periods

Color (ΔE_00_), lightness (ΔL'), chroma (ΔC'), and hue differences (ΔH’) are presented in Tables 1 and 2 for the enamel/dentin and composite resin groups, respectively. The L*a*b* standard averages (L= 92.38; a= 1.07; and b= 26.22) were used as references for the comparisons in enamel/dentin groups and periods, whereas the L*a*b* standard averages (L= 85.95; a= 0.12; and b= 26.42) were used as references for the comparisons in composite resin (groups and periods). Therefore, the comparison of these differences among groups and periods can also be seen in Figure 2 (for enamel/dentin groups) and Figure 3 (for composite resin groups). In addition, the WI_D_ values of the groups and periods may be seen in Table 3 and Figure 4.

**Table 1:** Color (ΔE00), lightness (ΔL’), chroma (ΔC’) and hue differences (ΔH’) for enamel/dentin groups and periods

	Original MTA				MTA Repair HP				NeoMTA Plus				Biodentine			
	ΔE_**00**_	ΔL’	ΔC’	ΔH’	ΔE_**00**_	ΔL’	ΔC’	ΔH’	ΔE_**00**_	ΔL’	ΔC’	ΔH’	ΔE_**00**_	ΔL’	ΔC’	ΔH’
7 days	2.2	-1.4	1.0	0.5	1.8	0.8	-0.3	0.4	2.0	1.1	0.0	-0.7	1.9	0.3	-2.1	-0.3
	± 1.2	± 2.3	± 4.0	± 0.9	± 0.9	± 1.9	± 3.2	± 0.6	± 0.7	± 1.9	± 3.3	± 0.8	± 1.2	± 2.6	± 2.1	± 1.1
15 days	3.0	-2.0	-0.8	1.2	1.8	1.0	-0.2	0.9	2.0	-0.5	-1.1	0.1	2.5	-1.3	-2.8	0.5
	± 1.6	± 2.6	± 5.3	± 1.2	± 0.4	± 1.4	± 2.7	± 0.7	± 1.0	± 2.5	± 3.0	± 0.9	± 1.8	± 3.6	± 2.3	± 1.1
30 days	2.3	-2.4	-2.1	0.7	1.7	-0.4	-1.5	1.0	2.6	-0.8	-1.8	-0.1	3.1	-0.1	-3.2	0.2
	± 1.2	± 2.1	± 2.5	± 0.5	± 0.5	± 1.4	± 2.4	± 0.5	± 0.9	± 2.7	± 4.1	± 0.9	± 1.4	± 3.5	± 3.7	± 1.4
45 days	3.0	-3.0	-1.4	1.7	2.0	-2.0	-0.3	0.5	2.1	-1.1	-1.7	0.5	2.4	-1.0	-1.9	0.9
	± 1.6	± 3.3	± 1.7	± 1.0	± 0.8	± 2.0	± 2.5	± 0.8	± 1.1	± 2.1	± 3.1	± 1.0	± 1.4	± 2.2	± 4.4	± 0.8
90 days	3.5	-4.5	-1.1	0.8	1.7	-1.5	-1.0	0.1	2.4	-2.8	-1.7	-0.3	2.7	-2.1	-2.9	-0.3
	± 2.0	± 3.2	± 3.8	± 0.8	± 1.1	± 2.1	± 2.4	± 0.6	± 0.8	± 1.3	± 2.4	± 0.8	± 1.1	± 2.0	± 3.3	± 1.3
300 days	3.4	-4.8	-1.0	0.3	2.0	-0.7	-2.2	-0.3	2.3	-1.2	-0.6	-1.4	3.4	-2.8	-2.1	-1.6
	± 1.3	± 2.0	± 2.8	± 1.2	± 0.9	± 1.9	± 2.1	± 1.5	± 1.1	± 2.3	± 3.1	± 0.9	± 2.2	± 3.5	± 4.2	± 1.9
2 years	3.4	-4.7	-1.2	-1.0	3.0	-2.5	1.0	-0.6	4.0	-4.6	-3.3	-2.0	3.8	-4.3	0.0	-1.4
	± 1.1	± 1.7	± 2.3	± 1.3	± 1.8	± 4.3	± 2.7	± 1.2	± 1.3	± 2.1	± 2.6	± 0.8	± 1.6	± 2.5	± 5.2	± 1.5

**Table 2:** Color (ΔE00), lightness (ΔL’), chroma (ΔC’) and hue differences (ΔH’) for composite resin groups and periods

	Original MTA				MTA Repair HP				NeoMTA Plus				Biodentine			
	ΔE_**00**_	ΔL’	ΔC’	ΔH’	ΔE_**00**_	ΔL’	ΔC’	ΔH’	ΔE_**00**_	ΔL’	ΔC’	ΔH’	ΔE_**00**_	ΔL’	ΔC’	ΔH’
7 days	1.8	-0.4	-1.9	0.7	1.6	1.0	-0.2	0.8	1.6	-0.4	0.0	0.0	1.7	0.8	0.9	-0.3
	± 0.9	± 2.5	± 1.0	± 0.7	± 0.7	± 2.0	± 1.5	± 0.8	± 1.3	± 2.9	± 1.7	± 0.8	± 0.7	± 2.1	± 2.0	± 0.7
15 days	2.2	0.3	-3.0	0.8	2.4	1.5	-1.5	0.9	1.7	0.1	-1.2	0.4	1.5	0.4	0.0	-0.6
	± 0.7	± 2.5	± 1.1	± 0.6	± 1.3	± 2.9	± 2.0	± 1.6	± 1.3	± 1.3	± 1.8	± 1.0	± 0.6	± 1.8	± 2.3	± 0.6
30 days	2.7	1.7	-4.2	-0.3	3.0	4.0	-2.5	0.1	2.1	1.8	-1.3	-0.7	2.6	2.9	-0.7	-1.2
	± 0.8	± 2.2	± 1.3	± 0.8	± 1.2	± 2.3	± 1.2	± 0.8	± 1.1	± 1.1	± 1.4	± 0.6	± 1.3	± 2.6	± 2.0	± 0.9
45 days	3.5	-3.5	-4.7	0.3	1.9	-0.4	-3.0	0.3	1.8	1.9	-1.5	0.0	2.6	1.0	-1.8	-0.4
	± 0.7	± 1.9	± 1.2	± 0.6	± 0.6	± 1.6	± 1.2	± 0.8	± 0.7	± 2.1	± 0.8	± 0.7	± 1.2	± 3.7	± 2.6	± 0.5
90 days	3.8	-3.1	-5.8	-0.3	2.3	-0.4	-3.2	-0.6	2.2	0.1	-2.7	-1.2	2.6	-2.3	-2.7	-1.4
	± 1.3	± 2.8	± 1.2	± 0.7	± 0.6	± 2.1	± 2.4	± 0.5	± 0.6	± 2.3	± 1.1	± 0.7	± 1.2	± 2.3	± 1.5	± 0.7
300 days	3.8	-2.3	-5.2	-1.2	2.6	0.4	-3.5	-1.1	5.4	-8.1	-2.7	-0.7	2.6	-1.2	-1.9	-1.9
	± 1.0	± 3.6	± 1.1	± 0.3	± 0.8	± 2.7	± 1.3	± 0.8	± 2.1	± 3.0	± 1.7	± 0.6	± 1.1	± 2.7	± 2.2	± 1.0
2 years	3.4	-1.1	-5.4	-1.7	3.2	1.5	-3.7	-2.0	5.9	-8.0	-4.7	-1.3	3.2	-0.8	-2.5	-3.1
	± 0.7	± 2.3	± 1.3	± 0.7	± 0.7	± 2.6	± 1.8	± 0.9	± 1.9	± 2.9	± 2.3	± 1.0	± 1.4	± 1.4	± 2.1	± 1.4

For enamel/dentin groups, the two-way ANOVA found significant differences among groups and periods tested (*p*<0.05) when color differences were evaluated. The interactions between the groups × periods were not significant (*p*=0.65). The smallest color differences were found in the MTA Repair HP group (1.7∆E_00_) after 30 and 90 days. NeoMTA Plus had the greatest color differences (4.0∆E_00_). However, the multiple comparisons test showed statistical similarity for the Original MTA, NeoMTA Plus, and Biodentine groups (α=0.05). As previously mentioned, the greatest color differences were observed after two years (NeoMTA Plus - 4.0 ∆E_00_). They were statistically different from all other periods tested (α=0.05). Despite the multiple comparisons test showing similar statistical values among the other evaluated periods (7, 15, 30, 45, 90, and 300 days), an increasing trend in color differences over time was noted.

When lightness (ΔL’), chroma (ΔC›), and hue differences (ΔH›) were evaluated by the repeated-measures ANOVA, significant lightness differences were found for the groups and periods tested (*p*<0.05). Interactions between groups × periods were not significant (*p*=0.67) (Table 4). All groups had a significant reduction in lightness, especially after two years of analysis (*p*<0.05). The Original MTA group had the greatest reduction in lightness after two years, being different from MTA Repair HP, NeoMTA Plus, and Biodentine groups, which were statistically similar (α=0.05). After two years of analysis, the greatest lightness differences were observed but they were similar in the 90 and 300-day periods (α=0.05). Regarding the chroma differences, Original MTA and NeoMTA Plus groups were similar to the other groups tested (*p*=0.036). However, MTA Repair HP and Biodentine groups were different from each other (α=0.05). Although the periods of analysis are not significant (*p*=0.28), all groups presented an increase in chroma values, with a trend toward red (a+). Concerning the hue, all groups had a trend toward blue (b-). The differences found were significant among groups (*p*<0.05) and periods (*p*<0.05). Interactions between groups × periods were not significant (*p*=0.57). The Original MTA and MTA Repair HP groups had the smallest hue differences, with similar statistical behavior (α=0.05). The greatest variations were observed after two years but with statistical similarity at the 300-day period (α=0.05).

**Table 4:** Repeated measures ANOVA of lightness (∆L’), chroma (∆C’), and hue (∆H’) differences for groups and periods

Enamel/dentin groups													
		ΔL’				ΔC’				ΔH’			
Variation Factor	df	SS	MS	F	P	SS	MS	F	P	SS	MS	F	P
Groups	3	234.049	780.163	124.223	<0.001	90.864	302.880	288.028	0.03	569.582	1.898.606	1.685.522	<0.001
Periods	6	492.296	820.493	130.644	<0.001	77.963	129.938	123.567	0.28	1.450.247	2.417.078	2.145.804	<0.001
Groups x Periods	18	92.885	51.603	0.8217	0.67	174.268	96.815	0.92068	0.55	183.460	101.922	0.90483	0.57
Error	252	1582.65	62.804			2649.94	105.156			2.838.579	112.642		
Total	279	2401.88				2993.03				5.041.867			
Composite resin groups													
		ΔL’				ΔC’				ΔH’			
Variation Factor	df	SS	MS	F	P	SS	MS	F	P	SS	MS	F	P
Groups	3	353.113	1.177.045	1.805.629	<0.001	358.766	119.589	423.263	<0.001	510.712	1.702.372	252.568	<0.001
Periods	6	805.680	1.342.799	2.059.903	<0.001	428.529	71.422	252.784	<0.001	1.869.118	3.115.196	462.178	<0.001
Groups x Periods	18	855.954	475.530	729.480	<0.001	39.529	2.196	0.7773	0.72	180.112	100.062	14.845	0.09
Error	252	164.272	65.188			712.000	2.825			1.698.542	0.67402		
Total	279	365.747				1538.82				4.258.483			

For the composite resin specimens, groups, periods, and interactions between groups × periods were significant (*p*<0.05). The smallest color differences were observed after 15 days in the Biodentine group (1.5∆E_00_), and the greatest differences were found in the NeoMTA Plus group, after two years (5.9∆E_00_). The greatest color differences were observed (α=0.05) after 300 days and two years, when statistical similarity was found between Original MTA and NeoMTA Plus groups . Differences in lightness were affected by groups, periods, and interactions between groups × periods (*p*<0.05) (Table 4). The statistical similarity was observed between Original MTA and NeoMTA Plus groups (α=0.05) and MTA Repair HP and Biodentine groups (α=0.05), with a more evident lightness reduction for the NeoMTA Plus group. The lightness reduction was more evident at the most advanced periods (90 days, 300 days, and two years), which were statistically similar (α=0.05).

Groups and periods were statistically significant when chroma differences were analyzed (*p*<0.05). Statistical similarity was also observed between MTA Repair HP and NeoMTA Plus groups (α=0.05), with an increase in chroma for all groups, especially for the Original MTA group, with a tendency towards red (a+). Chroma increased over time, with statistical similarity at the 90-day, 300-day, and two-year periods (α=0.05). Regarding the hue differences, repeated-measures ANOVA identified a significant difference between the groups and periods evaluated (*p*<0.05). The greatest hue differences were found for the Biodentine group (-3.1∆H’) after two years. Tukey’s multiple comparisons test found these differences in the Biodentine group and identified statistical similarities among the other experimental groups. The greatest variations were found after two years; however, this period differed from all other periods tested (α=0.05).

Regarding the WI_D_ index, the greatest values were observed after 30 days for the Biodentine group (20.0 WI_D_ - enamel/dentin) and the MTA Repair HP group (19.6 WI_D_ - composite resin). It was possible to observe that WI_D_ values decreased over time in these groups, especially after two years (Table 3 and Figure 3). Regarding the enamel/dentin specimens, ANOVA identified differences among the tested periods (*p*<0.05). Groups and interactions between groups × periods did not show statistical differences (*p*=0.57 and *p*=0.85, respectively). The periods of 300 days and two years were statistically similar (α=0.05). The periods of 7, 15, 30, 45, and 90 days were also similar (α=0.05). When the composite resin specimens were analyzed, groups and periods were statistically significant (*p*<0.05). The Tukey’s test identified statistical similarity between Original MTA and MTA Repair HP groups, with the greatest WI_D_ values, as well as between the periods of 90, 300 days, and two years (α=0.05), and 7, 15, and 30 days (α=0.05).

## Discussion

This 2-year assessment *in vitro* study aimed to investigate the discoloration induced by four hCSCs (Original MTA, MTA Repair HP, NeoMTA Plus, and Biodentine) on the enamel/dentin structure and a composite resin restoration. According to the results, the null hypothesis was rejected since the different hCSCs changed the color, lightness, chroma, hue, and whiteness index of teeth and composite resin over time.

Although this study aimed to simulate the clinical use of different hCSCs, as a laboratory study, it has some limitations, such as the use of bovine teeth to assess discoloration. Human teeth are the most used substrate for dental research.^27,28^ However, large quantities of human teeth in proper conditions for use may be challenging to obtain, primarily due to ethical concerns.^27,28^ Many studies have already adopted human and bovine teeth to evaluate the potential discoloration induced by hCSCs.^4,7-10^ The similar results obtained by both models ensure the reliability of using bovine teeth in studies involving esthetics demand.^4,7,29,32-34^

In addition to the bovine teeth used to fabricate the enamel/dentin discs, composite resin specimens were fabricated and used to test the potential discoloration of hCSCs. Some studies assessed the pre-application of a bonding agent to dentin to prevent tooth discoloration caused by hCSCs.^20,21^ However, to our knowledge, no studies have assessed the discoloration promoted by these types of cement on composite resin. Amongst their wide range of clinical applications,^2^ hCSCs may be used as pulp-capping materials, coming into direct contact with resinous-based restorative materials. It should also be highlighted that there is a clinical trend towards performing vital pulp therapies and regenerative procedures in a single appointment. These treatment modalities may expose composite resins to hCSCs before they finish their setting reactions.^35^ For this reason, their potential discoloration effect on these resinous-based materials must be tested. Therefore, standardized cavities were made at the center of dental and composite resin discs to mimic a pulp-capping clinical scenario.

Regarding the colorimetric analysis methodology, in the present study, the color differences (∆E_00_) were estimated from laboratory measurements, and the CIEDE2000 color difference system was used to validate the results.^23^ The CIEDE2000 system allows a better adjustment compared to the CIE L*a*b* system, especially when slight visual variances need to be assessed, such as those observed during the dental clinical routine.^23^ To correctly extrapolate the interpretation of results obtained in laboratory studies to real life, they should be compared with color difference thresholds, such as perceptibility (PT) and acceptability (AT).^24^ PT corresponds to a situation where the observer notices a significant color difference between two distinct objects. Conversely, AT corresponds to a situation where a color difference is still acceptable.^24^

An essential revision to the ISO/TR 28642 Dentistry-Guidance on color measurement was performed by Paravina, et al.^36^(2015). The authors provided a 50:50% PT and 50:50% AT under simulated clinical conditions, using both the CIE L*a*b* and CIEDE 2000 systems.^36^ The threshold values reported for the CIEDE2000 system were 0.8 and 1.8 ΔE_00_ for PT and AT, respectively. In the present study, the color differences found for all tested hCSCs over time were visually perceptible (color differences >0.8), as proposed by Paravina, et al.^36^(2015). Based on their previous study,^36^ Paravina, et al.^24^ (2019) reclassified the mismatch types as moderately unacceptable (>1.8 to ≤3.6 ΔE_00_), clearly unacceptable (>3.6 to ≤5.4 ΔE_00_), and extremely unacceptable (>5.4 ΔE_00_). Once again, despite the present study being an *in vitro* evaluation, the values obtained were in accordance with this new classification, ranging from moderately unacceptable to unacceptable after two years of assessment, depending on the hCSC tested, for enamel/dentin and composite resin. However, it is valid to state that the NeoMTA Plus group had an extremely unacceptable color variation (ΔE_00_ =5.9) after two years in the composite resin specimens.

The color variation over time, regardless of the hCSCs tested and the substrate experimental condition, had greater significance after two years of assessments (Tables 1 and 2). Furthermore, this color variation did not follow an ascending pattern throughout the experiment.^37^ This fact may be related to the polychromatic nature of both substrates tested^38^ and the non-linear release of chemical compounds by the hCSCs during the different experimental periods.^39,40^ These two variables probably played a vital role in the esthetics behavior of the tested cements.^38-40^

As the precursor of the hCSCs, the Original MTA is a gold-standard material.^8-10^ Thus, in this laboratory experiment, it was used as a positive control. The Original MTA has Bi_2_O_3_ as radiopacifier.^1^ The high atomic number of Bi (Z = 83) ensures greater radiopacity to this hCSC, even at lower concentrations (20% by weight).^1^ However, this high atomic number has a deleterious effect on the hydration mechanism of MTA.^1^ After the cement powder is mixed with water, the Bi_2_O_3_ is incorporated into the hydrated phase of the cement. A structure composed of ettringite, monosulphate, and hydrated bismuth calcium silicate is formed, which dampers the microstructure of MTA and negatively affects the physical-chemical and biological properties of this cement.^1-3^ In addition, the remaining Bi_2_O_3_ may undergo oxy-reduction or oxidation, forming metallic bismuth and bismuth carbonate, respectively.^41-43^ According to several studies, the dark precipitate created after these two phenomena leads to tooth discoloration over time.^41-43^ We also highlight that pre-etching of the dentin surface with 37% phosphoric acid was performed during the enamel/dentin disc restoration with composite resin. The amino acids present in the exposed collagen fibrils of the dentin organic matrix contributed to the Bi_2_O_3_ destabilization, also leading to the dark precipitate deposition.^8-10^In the present *in vitro* study, after 2 years of assessment, the specimens of the Original MTA group had a moderately unacceptable color difference (ΔE_00_=3.4) for enamel/dentin and composite resin. Conversely, during the 300 days, this color difference was unacceptable (ΔE_00_=3.8) for the composite resin substrate.

The results of this study showed a stable colorimetric pattern for up to 90 days for all tested hCSCs, regardless of the substrate condition. This trend has disappeared throughout the experimental periods of analysis, reaching its peak at the 300 day-period, except for the Original MTA group. At the 90-day period, the specimens of the Original MTA group already had more significant color differences (ΔE_00_=3.5 - moderately unacceptable - enamel/dentin; and ΔE_00_=3.8 - unacceptable - composite resin) than the other hCSCs. It should be emphasized that only the Original MTA has Bi_2_O_3_ in its composition. This radiopacifier is a factor to be considered when discoloration is assessed in shorter-term studies.^10^ On the other hand, the replacement of Bi_2_O_3_ by other radiopacifiers to avoid tooth discoloration is not supported by experiments with more extended periods of analysis,^4,7^ as the other tested hCSCs in this study presented moderate and unacceptable color difference values compared to baseline values after 300 days and two years of assessment. Therefore, methodologies using more extended analysis periods are fundamental to obtaining reliable results with materials that require esthetical demand, such as hCSCs.

A significant reduction was observed regarding the lightness differences (ΔL’), especially after two years of analysis. For the enamel/dentin substrate, the Original MTA group had the greatest reduction in lightness after this period, followed by NeoMTA Plus and Biodentine groups. For the composite resin specimens, the lightness reduction was significantly more evident for the NeoMTA Plus group than the other experimental groups. The negative values observed after the two years for most experimental groups (except for the MTA Repair HP group – composite resin) mean the specimens have become darker over time, indicating a noticeable discoloration for both substrates. This might be related to the diffusion of chemical compounds released by the hCSCs through the tested substrates.^39^ The oxidation process over time of iron oxide (Fe_2_O_3_), a compound present in the formulation of Original MTA and Biodentine, leads to the formation of tetracalcium ferroaluminate (C4AF), which causes tooth discoloration,^39^ and may also explain the increase in chroma values, especially for the Original MTA group, with a trend toward red.

The present study assessed the enamel/dentin and composite resin darkening by the CIELAB-based whiteness index calculation (WI_D_).^25^ The WI_D_ values remained stable over time for all experimental groups. However, these values decreased for the Biodentine and MTA Repair HP groups after two years for both substrates. For the Biodentine group, the WI_D_ values were in accordance with the lightness differences (ΔL’) observed in this period. According to Ramos, et al.^44^(2016), lightness was the most affected parameter for Biodentine after one year of *in vitro* evaluations. It is essential to mention that reduction in lightness contributes to color difference (ΔE_00_).^4^ On the other hand, the MTA Repair HP group had a conflicting colorimetric behavior concerning this parameter, demonstrating how the mechanism of discoloration induced by hCSCs remains unknown.^7,45,46^

Despite the similarity in the main components of the tested hCSCs,^45,46^ the tested cements presented distinct colorimetric patterns over time for both substrates. Subtle differences in some chemical components of the tested cements suggest changes in their colorimetric behavior over time.^45,46^ This phenomenon demonstrated the multi-factorial nature of the discoloration induced by hCSCs.^45,46^ The findings in our study cover a gap in the current literature, especially for resinous materials. However, further studies assessing the discoloration potential of hCSCs are still needed to achieve proper esthetic needs for this class of repair biomaterials.

## Conclusions

Within the limits of a laboratory study, the following conclusions may be drawn:

1. The tested hydraulic calcium silicate-based cement induced significant changes in the colorimetric behavior of the dental structures and composite resin over time, especially lightness.
2. The presence of Bi_2_O_3_ as a radiopacifier must be considered when the discoloration potential of hydraulic calcium silicate-based cement is assessed in shorter-term studies.
3. The replacement of Bi_2_O_3_ by other radiopacifiers to reduce dental and composite resin discoloration is not supported by experiments with more extended periods of analysis.
